# Extracellular Vesicle-Derived Non-Coding RNAs: Key Mediators in Remodelling Heart Failure

**DOI:** 10.3390/cimb46090559

**Published:** 2024-08-27

**Authors:** Jiayi Zhao, Huang Huang

**Affiliations:** 1 Department of Cardiovascular Surgery, The First Affiliated Hospital of Nanchang University, Nanchang 330006, China; z10901002666@163.com; 2 Jiangxi Medical College, Nanchang University, Nanchang 330031, China

**Keywords:** extracellular vesicles, heart failure, non-coding RNA, exosome, biogenesis, engineered extracellular vesicles, microRNA

## Abstract

Heart failure (HF), a syndrome of persistent development of cardiac insufficiency due to various heart diseases, is a serious and lethal disease for which specific curative therapies are lacking and poses a severe burden on all aspects of global public health. Extracellular vesicles (EVs) are essential mediators of intercellular and interorgan communication, and are enclosed nanoscale vesicles carrying biomolecules such as RNA, DNA, and proteins. Recent studies have showed, among other things, that non-coding RNAs (ncRNAs), especially microRNAs (miRNAs), long ncRNAs (lncRNA), and circular RNAs (circRNAs) can be selectively sorted into EVs and modulate the pathophysiological processes of HF in recipient cells, acting on both healthy and diseased hearts, which makes them promising targets for the diagnosis and therapy of HF. This review aims to explore the mechanism of action of EV-ncRNAs in heart failure, with emphasis on the potential use of differentially expressed miRNAs and circRNAs as biomarkers of cardiovascular disease, and recent research advances in the diagnosis and treatment of heart failure. Finally, we focus on summarising the latest advances and challenges in engineering EVs for HF, providing novel concepts for the diagnosis and treatment of heart failure.

## 1. Introduction

Cardiovascular disease (CVD) is the leading cause of the worldwide disease burden on human health, with high morbidity, high mortality, poor quality of life, and other characteristics, gravely endangering human life and health [1,2]. According to recent surveys, heart failure (HF) affects more than 64 million people globally, accounts for a large part of global morbidity and mortality, and is the final outcome of most CVDs [3,4,5]. In recent years, due to factors such as population growth and ageing, its case mix has evolved, with a significant increase in HF in the younger age groups. The total number of patients continues to climb [6]. HF is secondary to impairment of cardiac function caused by a variety of factors and is closely related to apoptosis, autophagy, inflammation, and cardiomyocyte remodelling. Ventricular insufficiency caused by myocardial interstitial fibrosis, as well as cardiomyocyte loss through apoptosis and necrosis, are essential components of the etiology of HF [7,8]. Cardiac hypertrophy is an adaptation to haemodynamic overload, but sustained overload can also lead to HF [9,10]. Despite the effectiveness of current treatments, it still leads to hospitalisation and death in the majority of patients. Since it is not yet completely curable, it is critical to investigate and enhance promising molecules for the diagnosis and therapy of HF [7,11,12].

**Figure 1:**
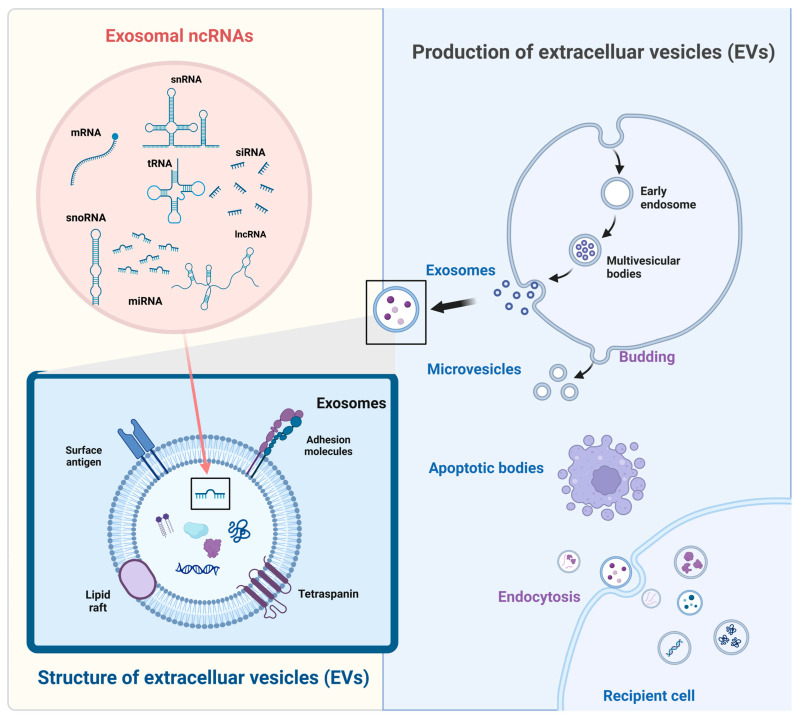
The main structure and classification of extracellular vesicles (EVs), the contents it carries such as ncRNAs. Mechanisms of EVs biogenesis and uptake.

Extracellular vesicles (EVs) are non-biological nanoparticles with diameters ranging from 40–1000 nm, containing a vast number of biologically active compounds within a lipid bilayer membrane, which are widely present in the circulatory system and various body fluids [13,14,15]. EVs can carry a wide range of biologically active inclusions, with non-coding RNAs (ncRNAs) being the most prominent of these [16]. ncRNA is a form of RNA that does not take part in translation but can alter normal gene expression and disease progression, including microRNAs (miRNAs), long ncRNAs (lncRNA), and circular RNAs (circRNAs) (Figure 1). Recent studies have shown that ncRNAs encapsulated in EVs are intriguing indicators that can play a part in the pathophysiology of various CVDs [17,18,19,20]. Due to its advantages of stability, easy accessibility, and modification, it can be a potentially therapeutically important target for HF in the future [21].

This paper will review the mechanisms of EV biogenesis and ncRNA sorting, focusing on the mechanisms by which extracellular vesicle-associated non-coding RNAs (EV-ncRNAs) regulate cardiac and vascular remodelling actions in HF, such as inflammation, fibrosis, angiogenesis, and oxidative stress, while also focusing on the use and targeting of EV-ncRNAs as diagnostic indicators and therapeutic tools for HF.

## 2. Size Classification of EVs, Generation Process, and Sorting Mechanism of EV-ncRNAs

### 2.1. Size Classification

EVs are lipid bilayer structures secreted by cells that carry physiologically active molecules, such as nucleic acids, proteins, lipids, and metabolites, and perform intercellular information transfer tasks [22,23,24,25]. These vesicles can be classed as exosomes, microvesicles, and apoptotic vesicles based on their creation method and size [14,21,22]. Exosomes, as one of the EVs, range in diameter from about 30 to 120 nm [15]. Microvesicles are another type of EV, containing ‘traditional’ microvesicles that range in diameter from 150 to 1000 nm and interact with target cells in a variety of ways, including cell fusion, phagocytosis, and specific responses. All cells generate both types of EVs, which are discharged into the extracellular environment and participate in intercellular communication as well as protein and RNA transport [26].

### 2.2. Biogenesis of the EVs

Exosomes are membranous nanoparticles that originate from the endosomal/multivesicular vesicle body system, while microvesicles are bigger EVs formed through plasma membrane outgrowth (Figure 1).

EVs generate microvesicles that bud directly from the plasma membrane, and microvesicle production needs rearrangement of the actin cytoskeleton to enhance plasma membrane expansion, followed by cleavage and release of the vesicles [27,28,29]. The small GTP-binding protein ADP-ribosylation factor 6 (ARF6) plays a key regulatory role in the formation of classical microvesicles. Activated ARF6 recruits ERK kinase to the cytoplasmic membrane by promoting the activity of phospholipase D. ERK is phosphorylated at the plasma membrane, which activates myosin light chain kinase (MLCK). Activated MLCK then phosphorylates myosin light chain (MLC) on the actin cytoskeleton and promotes actin contraction at the neck of microvesicles, which in turn leads to microvesicle shearing and release [30,31].

EVs produce exosomes that originate from the endosomal system, with early endosomes being intraluminal vesicles (ILVs) that invaginate and germinate from endosomal membranes, and late endosomes being multivesicular bodies (MVBs) that are further matured from ILVs [32,33]. Inward budding of late endosomal membranes forms ILVs within multivesicular endosomes (MVEs), reorienting endocytosed proteins to signal outwardly, and upon MVE fusion with the plasma membrane, releases these ILVs as exosomes into the extracellular space [34,35]. The endosomal sorting complexes required for transport (ESCRT) process is pivotal for sorting proteins into ILVs within MVEs and for MVB formation, involving both the ESCRT machinery and the endosomal ESCRT mechanism for efficient translocation and vesicle generation [32,34,36,37]. ESCRT is a cytoplasmic protein complex that sorts specific components into ILVs, precursors to exosomes, through the coordinated function of its four subcomplexes and auxiliary proteins. The non-endosome-dependent sorting mechanism complements ESCRT’s role by facilitating ILV and MVB formation via lipids, ceramides, tetraspanins, and heat-shock proteins, independent of the ESCRT machinery [38,39,40]. MVBs transport their contents towards lysosomal degradation or to the plasma membrane for exosome release, with SNAREs, Rabs, and Ras GTPases being crucial for MVB fusion with the target membrane [41]. The small GTPase ARF6 and its downstream effector phospholipase D2 (PLD2) are pivotal in regulating exosome biogenesis by controlling ILV to MVB outgrowth and EGFR degradation, with implications for MVB development and selective ESCRT-independent transport functions [32,37].

### 2.3. ncRNA Sorting

**Figure 2:**
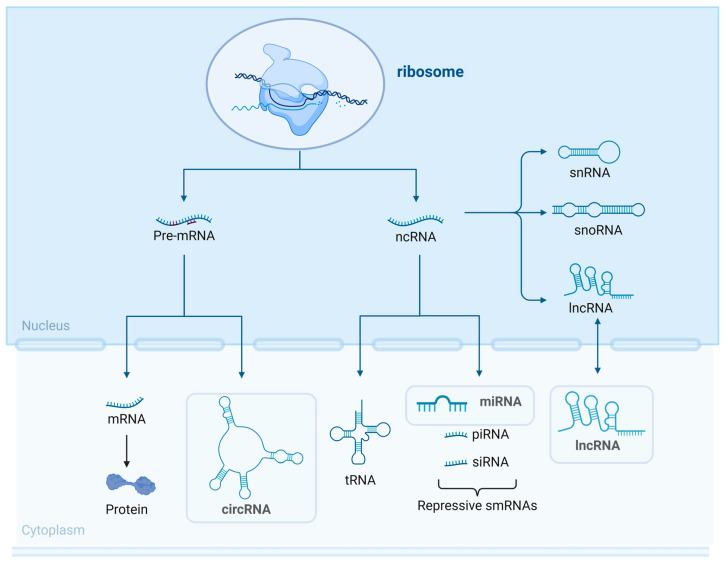
ncRNA classifications. RNAs are classified into two broad categories: messenger RNA (mRNA) and non-coding RNA (ncRNA).

Exosomes contain a variety of ncRNAs, such as miRNAs, lncRNAs, snRNAs, circRNAs, tRNAs, Y RNAs, vault RNAs, repetitive element RNAs, and fragment RNAs [16] (Figure 2). The loading of ncRNAs into exosomes is influenced by their biomass, size, and cellular location, with abundant cytoplasmic ncRNAs being more likely to be included, and RBPs playing a key role in their selective packaging [42]. RBPs such as hnRNP family members, YBX1, HuR, and others play a role in the selective loading of RNA species into EVs, potentially influencing post-transcriptional regulation and tissue homeostasis [16,29,42].

Exo-miRNAs are among the most abundant molecules in exosomes, and they play an important function in the control of gene expression. The sorting of miRNAs into exosomes is directed by four mechanisms: the sphingomyelinase 2-dependent secretion, hnRNPA2B1-mediated entry, miRISC complex formation with AGO2, and sequence-dependent 3′-terminal modifications that dictate intracellular or exosomal localization [43].

Despite their low cellular expression, circRNAs and lncRNAs are selectively enriched in EVs and can modulate miRNA activity, suggesting a role in transcriptional control through their extracellular distribution [44]. O’Grady et al.’s research indicates that EV packaging of mRNAs and lncRNAs is inversely related to their cellular abundance, with hNRNPA2B1 potentially playing a role in this selective sorting process for various RNA species [45]. Li et al. demonstrated that circRNA sorting of exosomes is at least partially regulated by changes in the levels of key miRNAs in the generating cells and that exo-circRNAs retain biological activity [46].

## 3. Participation Mechanisms

### 3.1. Oxidative Stress

Oxidative stress results from an imbalance in cellular redox balance, which has a significant impact on the progression of heart disease, particularly HF [20,47,48]. In the myocardium, increased reactive oxygen species (ROS) is a prominent molecular feature of HF [20]. Zhang et al. showed that the concentration of ROS has a dual effect on cellular function: low concentrations of ROS inhibit the lysosomal calcium channel TRPML1 and impact the fusion of MVBs with the lysosome, thereby increasing exosome production and secretion, whereas high concentrations of ROS activate autophagy and promote the fusion of MVBs with autophagosomes, leading to a decrease in exosome secretion [49]. Exosomes are important carriers of intercellular communications, and the small-molecule RNAs they carry, such as miRNAs, lncRNAs and circRNAs, can regulate the level of oxidative stress in recipient cells through specific signalling pathways [50,51,52].

miRNAs regulate heart function, including cardiac muscle contraction, cardiac development, and cardiac morphological alterations [53]. miRNAs can be strongly connected with oxidative stress-mediated cardiac remodelling in congestive HF through exosome-mediated deregulation of the Kelch-like ECH-related protein 1 (Keap1) nuclear factor erythroid 2-related factor 2 (Nrf2) pathway [54,55]. Tian et al. found that the regulation of Nrf2 in the myocardium after myocardial infarction is closely connected to oxidative stress and HF development, and that post-infarction-induced increases in local miRNAs may aggravate oxidative stress by inhibiting Nrf2 translation in HF [56]. The post-infarction-induced increase in local miRNAs may exacerbate oxidative stress by inhibiting Nrf2 translation in HF [57]. Cardiac miRNA-rich EVs from animals with congestive HF can promote oxidative stress and modulate sympathetic outflow by targeting the Nrf2/antioxidant signalling pathway. This hypothesis was tested in a study by Tian et al. that used in vitro-labelled circulating EVs and cardiac-specific membrane GFP transgenic mice to track the distribution of EVs of cardiac origin [51,58]. Specific miRNAs, such as miRNA-27a, have been found to be abundant in cardiac fibroblast-derived exosomes and contribute to oxidative stress [52]. Yan et al. found that mesenchymal stem cell-derived exosomes (MSC-Exos) block the NF-κB signalling pathway via miR-129-5p/TRAF3, reducing ventricular dysfunction, oxidative stress, apoptosis, inflammation, and cardiomyocyte fibrosis in HF rats [59].

lncRNAs can regulate mitochondrial oxidative stress by modulating the target miRNAs or proteins, affecting the progression of HF. lncRNAs maintain cardiac metabolic homeostasis and improve cardiac function by interacting with SIRT2 to activate the LKB1-AMPK signalling pathway [60]. Yan et al. [61] identified specific lncRNAs, such as lncRNA Caren, that can resist HF by inhibiting the ATM-DDR pathway, regulating translation of the Hint1 gene, and activating mitochondrial biogenesis [62]. Furthermore, the amount of lncRNA-NRF was positively correlated with the severity of HF following myocardial infarction [61].

circRNAs, a subset of non-coding RNAs, also contribute to the regulation of cardiac disease. For example, the mitochondria-localised circRNA Samd4 (circSamd4) reduces mitochondrial ROS production in cardiomyocytes after myocardial infarction, reduces cytotoxicity caused by oxidative DNA damage, and promotes cardiomyocyte repair [63].

### 3.2. Angiogenesis

Vascular endothelial cells (ECs) are a key cell type in angiogenesis, and they regulate vascular stability and permeability by secreting a range of bioactive chemicals. During the pathological process of heart failure, the function of ECs is severely affected, resulting in decreased angiogenesis and repair. ncRNAs in exosomes, particularly miRNAs, have a role in the angiogenic process by modulating EC gene expression and influencing proliferation, migration, and differentiation. For example, miR-126-5p was found to promote the proliferation and migration of ECs by inhibiting the expression of Notch1 repressor delta-like protein 1 (Dlk1) [64,65]. Furthermore, miRNAs such as miR-126, miR-26a, and miR-214 increase the expression of pro-angiogenic factors (VEGF) and promote angiogenesis by targeting and regulating certain genes [66]. Exo-ncRNAs may also enhance their role in angiogenesis through interactions with other bioactive molecules, e.g., miR-21 exerts its regulatory role in ECs by binding to the Ago2 complex and protecting its stability in the circulatory system [64].

Recent findings further highlight the role of exo-ncRNAs in regulating angiogenesis. For example, pro-inflammatory exosome-containing miR-155 is translocated to ECs to inhibit angiogenesis by downregulating its novel multiple target genes [67]. CVD conditions promote endothelial miR-92a-3p packaging into endothelial microvesicles (EMV) and regulate angiogenesis in receptor EC via a THBS1-dependent mechanism [68]. Under local ischaemia, hypoxia and inflammation after myocardial infarction, cardiomyocytes secrete EVs containing miRNAs such as miR-143 and miR-222, which regulate the proliferation and differentiation of ECs [69]. Furthermore, exosomes loaded with miR-21-5p improve cardiac healing by activating angiogenesis-related pathways and decreasing the proliferative ability of cardiomyocytes [70]. Duan et al. demonstrated that miR-214 may be a significant regulator of cardiac angiogenesis, controlling cardiac angiogenesis by modulating the expression of XBP1 [71].

### 3.3. Inflammation Response

In HF, the NF-κB and JAK/STAT pathways play a crucial role in cardiomyocyte apoptosis and cardiac remodelling. EV-miRNAs, such as miR-21 and miR-155, can modulate the activity of key genes in these pathways by targeting them [72,73].

Activation of NF-κB can increase the expression of inflammatory molecules including TNF-α and IL-6, worsening cardiac injury and fibrosis. MiR-21 is able to potentiate the activation of NF-κB by inhibiting negative regulators in the NF-κB pathway, which promotes the inflammatory response and cardiac injury [72]. Cytokine signalling is heavily reliant on the JAK/STAT pathway. In HF, its activation can lead to cardiomyocyte apoptosis and cardiac remodelling. miR-155 can reduce the inhibitory effect of suppressor of cytokine signalling 5 (SOCS5) by targeting it, thereby enhancing the activation of the JAK/STAT pathway and promoting inflammatory responses and apoptosis in cardiomyocytes [73].

In addition, inflammatory factors including TNF-α and IL-6 contribute significantly to the etiology of HF. EVs can regulate the expression of these inflammatory factors by carrying their mRNAs or miRNAs. For example, miR-155 can affect the inflammatory response of cardiomyocytes by regulating the expression of TNF-α [74,75]. Additionally, upregulation of miR-146a and miR-223 can reduce anti-tumour immune responses and protect cardiomyocytes from apoptosis by regulating the expression of inflammatory factors [69].

### 3.4. Autophagy and Apoptosis

Autophagy is an internal breakdown mechanism that helps to maintain cellular homeostasis and reduce stress [69]. In the context of HF, the adaptive role of autophagy can prevent or alleviate HF symptoms. It has been shown that autophagy helps cardiomyocytes adapt to hypoxic and nutrient-poor environments by removing damaged organelles and misfolded proteins, thereby reducing infarct size in situations such as myocardial infarction [76,77]. However, nonadaptive autophagy could be one of the potential causes of HF pathology. EVs secreted by the heart are strongly associated with the biological process of autophagy, and not only is the biogenesis of EVs related to autophagy, but autophagy is also regulated by EVs [78]. EVs contain ncRNAs, such as miR-30d, that can act as biomarkers of cardiomyocyte apoptosis and left ventricular remodelling, ameliorating apoptosis and exerting acute protection by targeting specific proteins such as MAP4K4 [79,80].

Apoptosis is a type of programmed cell death that contributes significantly to the development of HF. EV-ncRNAs, especially miRNAs such as miR-155-5p, can promote a pro-inflammatory phenotype and exacerbate myocardial injury through activation of the JAK2/STAT1 signalling pathway [81]. Furthermore, the deacetylase Sirt1 during apoptosis improves cardiac function and reduces cardiomyocyte apoptosis via the NF-κB p65/miR-155/BDNF signalling cascade response [82,83]. circRNAs act by acting as sponges for miRNAs, e.g., circRNA CDR1as is able to adsorb miR-135a and miR-135b and regulate cardiomyocyte apoptosis by affecting HMOX1 expression [78,84].

### 3.5. Immunomodulation

EVs secreted by cardiac stromal/progenitor cells selectively affect regulatory T cells (Treg), promoting improvements in their phenotype, activity, and proliferation [85]. Treg cells interacting with EVs show quicker proliferation, higher IL-10 production, and polarization towards an intermediate FOXP3+ RORγt+ phenotype. In a model of experimental autoimmune myocarditis (EAM), the application of EVs attenuated inflammation and functional decline of the heart, which was associated with an increase in the number of IL10+ Treg cells in the spleen. This finding suggests that regulation of T cells by EVs may provide a novel strategy for the treatment of inflammatory diseases [86].

The immunomodulatory role of B cells in HF cannot be ignored. Cardiac stromal/progenitor cell-secreted EVs have potential regulatory effects on B-cell function [87]. Although there are fewer preclinical studies on how B-cell EVs regulate macrophage polarisation, it is known that B-cell-derived EVs carry the MHC-II complex as well as co-stimulatory and adhesion molecules, and have an antigen-presenting capacity similar to that of primary B cells. These EVs are capable of delivering different types of antigens and thus may influence different types of immune responses. In addition, B cells may directly inhibit the polarisation of M2-type macrophages through their EVs, thereby increasing myocardial inflammation [88].

Natural killer cells (NK cells) serve a role in identifying and destroying target cells under stress. They are also engaged in the regulation of the immune response, particularly in the regulation of T-cell activities [72,89,90]. It has been shown that resting and activated human NK cells and their secreted EVs contain specific microRNA libraries that specifically target molecules involved in the Th1 response, such as miR-10b-5p, miR-92a-3p, and miR-155-5p. NK-EV promotes down-regulation of GATA3 mRNA and subsequent de-repression of TBX21 in CD4 T cells, leading to Th1 polarisation and the production of IFN-γ and IL-2 [89]. Furthermore, NK-EVs influence the activity of Monocyte-derived Dendritic cells (Mo-DC), activating them and improving their presentation and co-stimulatory capabilities [91].

Macrophages are important antigen-presenting cells capable of releasing EVs carrying functional cargoes. M1-type macrophages are associated with inflammation, whereas activation of M2-type macrophages indicates the subsidence of inflammation and the onset of tissue regeneration. Studies have shown that human M1 and M2 primary macrophages release EVs with different RNA cargoes, which may contribute to the unique influence of these cell subpopulations on their microenvironments [67,92]. In particular, miR-192-5p can activate M1 macrophages by regulating Rictor/Akt/FoxO1 signalling [93]. A rat model of myocardial infarction showed an increase in circulating EVs carrying pro-inflammatory cytokines such IL-1α, IL-1β, and RANTES. Reducing these pro-inflammatory EVs improved heart function [94].

### 3.6. Fibrosis

Cardiac fibrosis is a fundamental pathogenic process in HF, characterized by extracellular matrix remodelling and aberrant collagen buildup. Cardiac fibroblasts play an important role in this process, and their proliferation and activation lead to altered cardiac structure and impaired function. Numerous studies have shown that ncRNAs regulate cardiac fibrosis. Exo-ncRNAs from various sources contribute to the intricate regulation of all facets of cardiac fibrosis [95,96]. Because of exosomes’ ability in delivering ncRNAs, their involvement in regulating cardiac fibrosis is gaining attention.

Various ncRNAs govern cardiac fibroblast activation and proliferation during cardiac fibrosis. In particular, miRNAs carried by EVs, such as miR-494-3p, have been shown to exacerbate myocardial fibrosis by targeting the PTEN-AKT/Smad2/3/ERK signalling pathway and promoting the activation of cardiac fibroblasts [97]. Furthermore, genetically engineered human induced pluripotent stem cell (hiPS)-derived EVs harbouring miR-1 and miR-199a reduced apoptosis in cardiac fibroblasts, promoting cardiac fibrosis [98]. These findings highlight the possible role of EVs in cardiac fibrosis and offer novel avenues for future therapeutic treatments.

In addition to miRNAs, circRNAs play essential roles in cardiac fibrosis. For example, EVs containing circUbe3a were able to affect the proliferation and migration of cardiac fibroblasts, thereby exacerbating myocardial fibrosis [99]. Furthermore, overexpression of miR-208a in cardiomyocytes and their generated EVs increased fibroblast proliferation and differentiation, as well as the progression of cardiac fibrosis [100]. These findings highlight the complex regulatory role of ncRNAs carried by EVs in the development of cardiac fibrosis and may be a novel target for the treatment of HF.

EVs include ncRNAs that not only contribute to cardiac fibrosis but may also play an active role in cardio protection and repair. For example, exosomes containing LINC00636 inhibit MAPK1 by overexpressing miR-450a-2-3p, which ameliorates cardiac fibrosis [101]. Furthermore, upregulation of miR-29a in exosomes acts as an information conduit between cardiomyocytes and promotes antifibrotic effects to prevent ventricular dysfunction and HF [102]. These findings imply that by modulating ncRNAs in EVs, novel treatments for heart fibrosis and HF may become available.

## 4. Diagnosis and Prognosis

**Figure 3:**
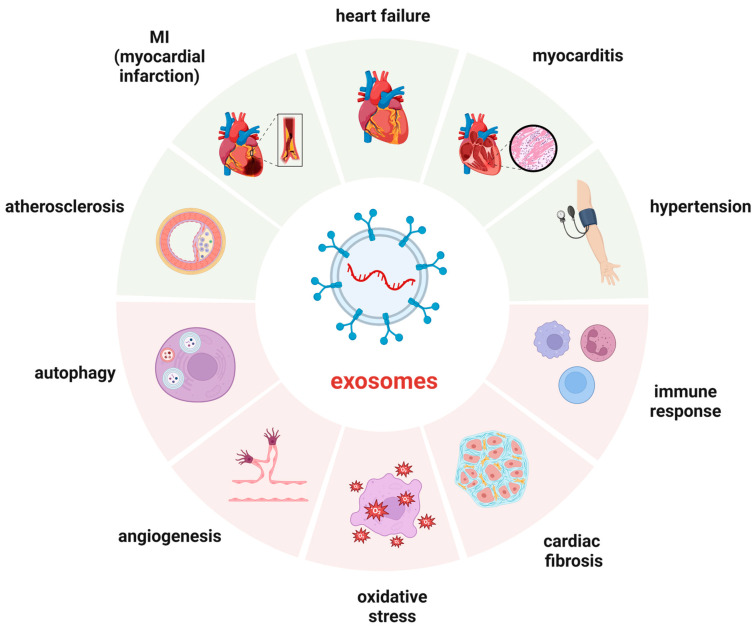
Clinical applications of drug-loaded exosomes and biogenesis and mechanism of action of exosomes. Engineered exosomes can deliver drugs or nucleic acids to target cells or tissues, modulating their biological functions and achieving therapeutic effects.

HF is a profound clinical condition characterized by the inability of the heart to pump blood effectively, resulting in inadequate blood supply to the body’s organs and tissues. The intricate pathophysiological mechanisms of HF involve a myriad of factors that contribute to the deterioration of cardiac function [7,8,9]. The heart’s pumping action is compromised in HF due to the stiffness or weakness of the cardiac muscle, a condition that can arise from various etiologies, including coronary artery disease, hypertension, valvular heart disease, and myocardial infarction (Figure 3). The subsequent remodelling of the heart leads to a decrease in the ejection fraction, the percentage of blood pumped out of the heart with each beat, which is a critical measure of heart function [3].

The diagnosis of HF typically relies on a combination of clinical signs, symptoms, imaging studies, and biochemical markers. The most commonly utilized biomarkers for the diagnosis and prognosis of HF are natriuretic peptide and cardiac troponin. Furthermore, next-generation biomarkers for screening, diagnosis, and prognostic assessment of HF, such as soluble sources of tumorigenicity 2 (sST2), galectin-3 (Gal-3), and growth differentiation factor-15 (GDF-15), have showed promising applications [103,104,105]. However, the diagnostic and prognostic value of the above markers that have been applied in clinical practice can be limited by the nature of the disease, the age of the patient, lifestyle, and many other factors. Therefore, the diagnosis and prognosis of HF also require new biomarkers that are more advantageous, non-invasive, and more sensitive than the traditional markers to assist in the accurate diagnosis and prognosis of HF. ncRNAs are stably present in body fluids and can be sensitively and specifically detected by specific amplification of their sequences. ncRNAs have been shown to be useful in the diagnosis and differential diagnosis of HF, the assessment of disease severity and prognosis, the risk of cardiovascular events after discharge from the hospital in HF patients, and patient individualization. ncRNA has demonstrated good potential in the diagnosis and differential diagnosis of HF, the assessment of disease severity and prognosis, the assessment of cardiovascular event risk in patients after discharge from hospitals, and the individualized medical management of patients [106].

### 4.1. Diagnosis of Heart Failure

The finding of altered levels of ncRNAs, which are released extracellularly and circulate in a conserved form in biological fluids in patients with HF, reveals the possibility of utilising circulating ncRNAs, especially EV-rich ncRNAs, as biomarkers of HF, facilitating the diagnosis of HF.

In recent years, many circulating EV-miRNAs are non-invasive prognostic and diagnostic indicators for HF [107,108]. Galluzzo et al. showed that six miRNAs (miR-210-3p, miR-22-5p, miR-22-3p, miR-21-3p, miR-339-3p, and miR-125a-5p) were significantly correlated with the HF biomarker [16,109]. Ding et al. revealed that miR-21-5p, miR-30a-3p, miR-30a-5p, miR-155-5p, miR-216a, and miR-217 may be new diagnostic biomarkers for HF and related CVD [107]. Several circulating EV-miRNAs, including miR-92-5p, miR-146a, miR-181c, and miR-495, have demonstrated diagnostic potential for HF [110]. In addition, two circulating EV-rich miRNAs associated with HF, including miR-30d-5p and miR-126a-5p, were downregulated in the circulating EV and left ventricle. This consistently correlates with decreased cardiac output and has the potential to serve as a biomarker of heart failure with preserved ejection fraction (HFpEF) in patients [110,111,112]. Li et al. evaluated the diagnostic value of circulating miR-302s in acute heart failure (AHF) and showed that circulating miR-302s, especially miR-302b-3p, had high accuracy in discriminating HF from non-HF patients, and that the combined detection of miR-302b-3p and NT-proBNP improved the accuracy of AHF diagnosis [113]. Wu et al. found that exo-miR-92b-5p had high sensitivity and specificity for the diagnosis of HFrEF, and serum levels of exo-miR-92b-5p could be used as a diagnostic marker for HFrEF [114]. Goren et al. isolated miRNAs from serum exosomes and quantified 186 miRNAs. The study showed that miR-423-5p had good diagnostic accuracy for HF, and combined detection of miR-423-5p, miR-320a, miR-22, and miR-92b identified patients with systolic HF and correlated with important clinical prognostic parameters [115]. miR-423-5p can be used as a biomarker for the diagnosis of HF and has more significant advantages compared to other partial miRNAs, but the gap to B-type natriuretic peptide (BNP) is still large. Presumably, the combination of miRNA and BNP is a better detection method [116].

### 4.2. Prognosis of Heart Failure

ncRNAs, especially miRNAs, carried by EVs play an important role in the prognosis of HF. These small RNA molecules are involved in the onset and progression of heart disease by regulating gene expression and are considered as promising biomarkers due to their stability and ease of detection in body fluids.

HF is a serious clinical condition whose prognosis is influenced by a variety of factors. In recent years, ncRNAs carried by EVs have been found to be valuable in the prognostic assessment of HF (Table 1). miRNAs such as miR-192, miR-34a, miR-425, and miR-744 are considered promising prognostic biomarkers for HF due to their enrichment in EVs [110,117]. These miRNAs are involved in the regulation of cardiac function and the progression of CVD by modulating gene expression in cardiac tissues.

In patients with advanced HF, specific miRNAs, such as miR-125a-5p, miR-10b-5p, and miR-9-5p, have been found to be associated with poor clinical outcomes. The levels of these miRNAs were associated with cardiac death, cardiac transplantation, or the need for mechanical circulatory support, and they added significant prognostic value to the Barcelona Bio-HF scoring system. In addition, a study by Sardu et al. found that downregulation of miR-130a-5p was associated with poor prognosis in response to endothelial dysfunction (ED) and cardiac resynchronisation therapy (CRTd), suggesting that serum miR-130a-5p may serve as a new biomarker of prognosis in HF [118].

Other studies have identified a potential role for EV-miRNAs in the prognosis of HF. For example, Seronde et al. found that low plasma levels of miR-423-5p on admission were associated with poor long-term prognosis in patients with AHF [119,120]. In addition, a study of 496 patients with AHF by van Boven et al. showed that significant upregulation of serum miR-1306-5p levels was independently and positively associated with adverse clinical outcomes in AHF [121]. These findings highlight the potential of EV-derived miRNAs as a prognostic assessment tool for heart failure and may contribute to the development of new diagnostic and therapeutic strategies [117]. EV-rich miRNAs, such as miR-192, miR-34a, miR-425, and miR-744, show promise as predictive biomarkers for HF.

**Table 1:** Some EV-ncRNAs that can be used as potential therapeutic targets.

Exosomal Cargo	Functional Mechanism	Cell Source	Functions	Ref.
miR-21a-5p	miR-21a-5p/ITGAV/Col1α	FPC	Reduces PO-induced cardiac fibrosis and improves cardiac function	[122]
miR-126	TGF-β/Smad3	EPC	Improves cardiac fibrosis	[123]
miR-9-5p	VPO1/ERK	iPSC	Protects against Dox-induced cardiomyopathy	[124]
miR-125a-5p	Klf13, Tgfbr1, Daam1	MSC	Alleviates myocardial ischemia/reperfusion (I/R) injury	[125]
miRNA-205	metalloproteinase-3	ADSC	Reduces myocardial fibrosis, inhibits myocardial apoptosis, promote angiogenesis	[126]
miR-22-3p	FURIN	CM	Increases the risk of HF damage	[127]
miR-129-5p	TRAF3, NF-κB	MSC	Protects the heart from HF	[59]
miR-195-3p	PTEN/AKT	CF	Promotes cardiac fibrosis and dysfunction after MI	[128]
miR-98-5p	TLR4, PI3K/Akt	BMSC	Protects against MI/RI	[129]
miR-217	PTEN	CM	Therapeutic target for CHF	[130]
circ_LAS1L	circ_LAS1L/miR-125b/SFRP5	CF	Regulates myocardial fibrosis after MI	[131]
circ_BMP2K	miR-455-3p, SUMO1	CF	Regulates myocardial fibrosis	[132]
circ_NSD1	miR-429-3p, Wnt/β-catenin	CF	Treatment of cardiac fibrosis	[133]
circ_0023461	miR-370-3p/PDE4D	AC16	Silencing protects cardiomyocytes from hypoxia-induced dysfunction	[134]
circ_0097435	Sponging multiple miRNAs	DOX-treated CM	Influences HF	[135]

## 5. Engineered EVs for HF Therapy

### 5.1. Self-Derived ncRNA Therapy

EV-ncRNAs have been widely investigated for the treatment of HF. Several miRNAs, including miR-126, miR-146a, miR-125a-5p, miR-125b-5p, miR-29b, miR-98-5p, miR-30e, and miR-30d, have been shown to preserve cardiac functioning [21,110,136]. The distribution of these miRNAs via EVs indicates fascinating clinical applications in HF therapy [137]. For example, EV-rich miR-205 improves adipose tissue-derived mesenchymal stem cell (ADSC)-derived EVs in acute myocardial infarction-induced heart damage, implying therapeutic potential [110,126]. Furthermore, EVs secreted from other stem cells, including human cardiac progenitor cells (hCPC), mesenchymal stem cells (MSCs), and induced pluripotent stem cells (iPSCs), demonstrated angiogenic and cardioprotective properties in a rodent model of myocardial infarction by significantly increasing endothelial cell proliferation, migration, and tube formation [21,138,139]. EV-miRNA research also revealed that several angiogenic and cardioprotective miRNAs, including as miR-210, miR-126, and miR-17–92, were overexpressed in these stem cell-derived EVs. For instance, suppressing miR-210 in MSC-EV dramatically reduced proangiogenesis in vitro and in a mouse model of myocardial infarction [140]. Recently, human umbilical cord MSC-derived EVs preloaded with miR-29b mimics also showed potent antifibrotic activity to prevent excessive cardiac fibrosis after myocardial infarction [21,110,141,142].

In addition to miRNAs, other ncRNAs, such as lncRNAs and circRNAs, should be considered in HF treatment. For example, lncRNAs are abundantly expressed in myocardial tissues and are engaged in the control and regulation of genes in cardiomyocytes, which can affect cardiomyocyte physiological activities by targeting and play an important part in the development of ischemic heart disease (IHD). lncRNAs may represent a novel target for treating ischemic heart disease by regulating the processes of cardiomyocyte proliferation, differentiation, and maturation, as well as the response to inflammation and oxidative stress. The lncRNA HOTAIR was found to be reduced in a mouse model of HF, whereas overexpression of HOTAIR improved cardiac function through a mechanism that may involve interaction with miR-30a-5p, which in turn affects the expression of KDM3A and BNIP3, thereby improving oxidative stress and inflammatory responses [143].

These findings emphasize the promise of modified EVs in HF treatment, notably in controlling cardiac cell activity and the cardiac microenvironment via ncRNAs. More effective and personalized therapy options for HF are predicted to be developed in the future by carefully adjusting miRNA load in EVs. To achieve this, in-depth research on EV formation, miRNA activity, and the efficiency and safety of their distribution in vivo are required to assure the therapy’s viability and efficacy. Meanwhile, the inclusion of lncRNAs and circRNAs offers new ideas and tactics for HF treatment, although more research is needed to investigate their unique roles and processes in HF therapy.

### 5.2. Combined and Targeted Drug Delivery

Engineered EVs can be used not only to deliver ncRNAs, but also to combine and target the delivery of drugs to enhance the efficacy of HF therapy. By combining drugs with EVs, drug targeting can be improved and side effects reduced [144]. Targeting EVs secreted from damaged hearts by exogenously preloading miRNA inhibitors into EVs, such as miR-27a, miR-28, and miR-34a, may represent another therapeutic strategy [110]. In addition, cardiac function after myocardial infarction was effectively restored by designing EVs with cardiac homing peptides and delivering miRNA-21 using genetic therapy. Pei et al. identified that bone marrow mesenchymal stem cells (BM-MSC) under hypoxic conditions secrete EVs enriched with miRNA-125b-5p. When miR-125-enriched EVs were combined with ischemic myocardial targeting peptides and delivered intravenously in a mouse model of acute myocardial infarction, cardioprotective miRNAs were highly selective for the ischemic myocardium [110,145].

### 5.3. Current Limitations and Challenges of Treatment

Although engineered EVs show great potential in HF therapy, they still face a number of technical and clinical challenges [122]. Firstly, quantification techniques for EVs, such as dynamic light scattering, nanoparticle tracking analysis (NTA), and resistive pulse sensing (RPS), despite being widely used, are limited in the robustness and comparability of their measurements due to a lack of standardisation [122]. The limitation of these techniques is that they typically measure all particles and cannot distinguish between small exosomes (sEVs) and other particles such as lipoprotein particles, protein aggregates, EV aggregates, or other contaminants. This may result in less pure samples incorrectly showing higher levels of EVs [146]. Moreover, the process of isolation and purification of EVs presents challenges. For instance, the use of ultracentrifugation, while allowing for the separation of EVs, may introduce bias in the sample handling process [147]. To improve the purity and yield of EVs, it is recommended to use additional measurements such as total protein and/or lipid content, or to quantify EV-tagged proteins by enzyme-linked immunosorbent assay (ELISA) or protein blotting (semi-quantitative) [148].

In terms of clinical applications, the biocompatibility and immunogenicity of EVs are key considerations. Despite the low immunogenicity of EVs, they may still trigger an immune response under certain circumstances, especially in EVs of heterologous origin [149]. In addition, the stability and storage conditions of EVs are crucial for their viability in clinical applications. Studies have shown that EVs may undergo degradation or loss of function under different storage conditions, which requires further studies to determine the optimal storage and transport conditions [150]. In addition, the targeting and delivery efficiency of EVs are key to realising their application in HF therapy. Improving the targeting and delivery efficiency of EVs, especially in HF therapy, requires further research and optimization [150].

Finally, translating EVs from laboratory studies to clinical applications requires overcoming many regulatory and ethical hurdles. Ensuring the safety, efficacy, and quality control of EVs is a critical step in conducting clinical trials and obtaining regulatory approval [151]. Despite these challenges, the use of EVs in HF therapy remains promising, and future research needs to focus on improving the purity, standardisation, biocompatibility, stability, targeting, and delivery efficiency of EVs, as well as addressing clinical trial and regulatory concerns.

## 6. Conclusions

In conclusion, the intricate regulatory network of extracellular vesicle (EV)-derived non-coding RNAs (ncRNAs) in heart failure (HF) presents a multifaceted approach to both understanding and treating this complex disease. The capacity of EVs to transport ncRNAs, including miRNAs, lncRNAs, and circRNAs, offers a novel avenue for targeted therapies, with potential to modulate key pathophysiological processes such as inflammation, fibrosis, and angiogenesis. The identification of specific ncRNAs as diagnostic and prognostic biomarkers further underscores the transformative impact of EVs in cardiac medicine. Despite the challenges in standardization, biocompatibility, and clinical translation, the future of HF management appears poised for a paradigm shift, with engineered EVs leading the way in personalized and precision medicine. As our comprehension of the underlying mechanisms deepens, so does our optimism for the development of effective, safe, and tailored treatments for patients with HF.
